# Does supplemental private health insurance impact health care utilization and seeking behavior of residents covered by social health insurance? Evidence from China National Health Services Survey

**DOI:** 10.1186/s12939-024-02158-8

**Published:** 2024-05-31

**Authors:** Fengsai Bie, Xiaoling Yan, Wuqi Qiu, Ayan Mao, Yueli Meng, Min Cai, Renke Yang, Yaoguang Zhang

**Affiliations:** 1 National Center for Occupational Safety and Health, National Health Commission of the People’s Republic of China, Beijing, 102308 China; 2 Institute of Medical Information, Chinese Academy of Medical Sciences and Peking Union Medical College, Beijing, 100020 China; 3 Center for Health Statistics and Information, National Health Commission, Beijing, 100044 China

**Keywords:** Private health insurance, Social health insurance, Health care seeking behavior, Health care utilization, Choice of health care provider

## Abstract

**Background:**

Supplemental private health insurance (PHI) plays a crucial role in complementing China’s social health insurance (SHI). However, the effectiveness of incorporating PHI as supplementary coverage lacks conclusive evidence regarding its impact on healthcare utilization and seeking behavior among SHI-covered individuals. Therefore, investigating the effects of supplementary PHI on health care utilization and seeking behavior of residents covered by social health insurance is essential to provide empirical evidence for informed decision-making within the Chinese healthcare system.

**Methods:**

Data from the 2018 China National Health Services Survey were analyzed to compare outpatient and inpatient healthcare utilization and choices between PHI purchasers and non-purchasers across three SHI schemes: urban employee-based basic medical insurance (UEBMI), urban resident-based basic medical insurance (URBMI), and the new rural cooperative medical scheme (NRCMS). Using the Andersen Healthcare Services Utilization Behavior Model as the theoretical framework,binary logistic regression and multinomial logistic regression (MNL) models were employed to assess the impact of PHI on healthcare utilization and provider preferences.

**Results:**

Among UEBMI, URBMI, and NRCMS participants with PHI, outpatient visit rates were 17.9, 19.8, and 21.7%, and inpatient admission rates were 12.4, 9.9, and 12.9%, respectively. Participants without PHI exhibited higher rates for outpatient visits (23.6, 24.3, and 25.6%) and inpatient admissions (15.2, 12.8, and 14.5%). Binomial logistic regression analyses revealed a higher probability of outpatient visits and inpatient admissions among UEBMI participants with PHI (*p* < 0.05). NRCMS participants with PHI showed a lower probability of outpatient visits but a higher probability of inpatient admissions (p < 0.05). Multinomial logistic regression indicated that NRCMS participants with PHI were more likely to choose higher-level hospitals, with a 17% increase for county hospitals and 27% for provincial or higher-level hospitals compared to primary care facilities.

**Conclusion:**

The findings indicate that the possession of PHI correlated with increased utilization of outpatient and inpatient healthcare services among participants covered by UEBMI. Moreover, for participants under the NRCMS, the presence of PHI is linked to a proclivity for seeking outpatient care at higher-level hospitals and heightened utilization of inpatient services. These results underscore the nuanced influence of supplementary PHI on healthcare-seeking behavior, emphasizing variations across individuals covered by distinct SHI schemes.

**Supplementary Information:**

The online version contains supplementary material available at 10.1186/s12939-024-02158-8.

## Background

China has sequentially introduced three distinct social health insurance schemes, namely the urban employee-based basic medical insurance scheme (UEBMI, initiated in 1997), the new rural cooperative medical scheme (NRCMS, initiated in 2003), and the urban resident-based basic medical insurance scheme (URBMI, initiated in 2007), each targeting different demographic groups. The allocation of citizens to specific insurance schemes is contingent upon their census register and employment status. As of the conclusion of 2011, had coverage under these three basic medical insurance schemes, maintaining a coverage rate exceeding 95% since then [1]. Despite this widespread coverage, the robustness of health security remains relatively limited, characterized by introductory, co-payment, and ceiling parameters for outpatient and inpatient services. These parameters vary across different demographic groups and hospital tiers, resulting in incomplete coverage that fails to fully address the medical protection needs of Chinese residents. The Rural Health Security Survey Report released by the Chinese Academy of Social Sciences in 2020 highlighted that even with full coverage of basic medical insurance in rural areas, approximately 30% of rural residents perceive their personal medical insurance level as insufficient, necessitating the supplementary role of private health insurance (PHI) [2].

Since the year 2000, developed countries have progressively incorporated supplementary health insurance, namely private health insurance, into their healthcare systems [3, 4]. In China, the development of PHI as supplementary medical insurance has witnessed rapid growth, with premium income and its share in personal insurance escalating annually. Premiums increased nearly 13 times from 2007 to 2018, and the share augmented by 12.4 percentage points [5]. Favorable policy environments further support PHI development, positioning it as a supplement to basic medical insurance. (Appendix 1) According to the 2018 National Health Service Survey report, the proportion of Chinese residents purchasing PHI increased from 6.9% in 2008 to 13.6% in 2018 [6, 7].

In the universal healthcare coverage system, the theory of moral hazard suggests that private health insurance, acting as supplementary medical insurance, may lead to excessive utilization of healthcare services. This implies that under the protection of PHI, individuals often tend to seek more medical services, thus intensifying the pressure on healthcare costs and affecting the rational allocation of overall medical resources. Such individual behavior brings negative consequences to society as a whole, potentially resulting in inequitable distribution of social resources, a crisis of trust, and a decline in public confidence in the insurance industry, among other public moral hazards [8, 9]. Furthermore, the social health insurance system bears some responsibility for the increased service utilization caused by PHI, known as the financial spillover effect of PHI on public health insurance [10].

Several studies have indicated that private health insurance (PHI) is associated with an increased utilization of health services. Specifically, some research has found that individuals with dual health insurance coverage are more likely to utilize public health systems [11]. Additionally, in certain countries, the combination of mandatory occupation-based health schemes with voluntary private insurance, known as dual coverage, has a positive impact on healthcare utilization rates [12–14]. Furthermore, research has found a significant positive correlation between the addition of supplemental health insurance to compulsory public insurance and healthcare utilization [15, 16]. However, not all studies support the idea that PHI promotes the utilization of medical services [2, 17].

Private health insurance significantly impacts individuals’ healthcare provider selection behavior, particularly among those covered by social health insurance. Studies across multiple countries show that individuals with PHI tend to visit specialists more frequently, indicating a preference for specialized care [18–21]. However, findings vary in some countries like France, Belgium, and Ireland, where complementary PHI doesn’t affect the choice between specialists and general practitioners [22–24]. In China, individuals with PHI and both urban and rural health insurance are more likely to choose high-level medical institutions for treatment [2]. Overall, PHI not only influences individuals’ preference for specialist care but also affects their choice of higher-level medical facilities, especially in countries with diverse healthcare systems and insurance coverage arrangements.

The relationship between Private Health Insurance (PHI) and other health insurances is complex and varied. Research indicates that the presence of PHI is closely associated with an increase in healthcare service utilization, particularly among individuals with dual health insurance coverage. Furthermore, in certain countries, the combination of mandatory occupational health schemes with voluntary private insurance, known as dual coverage, has been shown to enhance healthcare utilization rates. Additionally, the addition of supplemental health insurance to compulsory public insurance has also been positively correlated with healthcare utilization rates. However, not all studies support the notion that PHI promotes healthcare service utilization. Moreover, PHI not only influences individuals’ selection of healthcare providers, especially among those covered by social health insurance, but also affects their preference for specialist care. Cross-national studies demonstrate that individuals with PHI tend to visit specialists more frequently, though in some countries, supplemental PHI does not impact the choice between specialist and general practitioners. In China, the likelihood of individuals choosing high-level medical institutions for treatment is also associated with PHI. Overall, PHI not only influences individuals’ preference for specialist care but also impacts healthcare service selection in countries with diverse healthcare systems and insurance coverage arrangements. This research utilizes cross-sectional data from China’s National Health Services Survey (CNHSS) conducted in September 2018 to examine the impact of PHI on outpatient and inpatient service utilization and hospital choice among people covered by Social Health Insurance (SHI). The outcomes of this study are expected to provide additional empirical evidence and serve as a reference for decision-making regarding the formulation and implementation of supplementary PHI policies, both within China and internationally.

## Methods

### Survey design

The CNHSS Survey, initiated in 1993, is conducted at five-year intervals, employing a nationwide sample approach (94 sample counties for the first four iterations and 156 sample counties and districts for the fifth and sixth iterations). Recognized as the largest household health inquiry survey in China. In terms of survey methodology, the Sixth National Health Service Survey implemented measures for quality control. This included the development of the survey plan and survey guidelines, which stipulated that trained surveyors must achieve 100% technical consistency. The completion rate of the survey was required to be over 95%. The survey emphasized that respondents should answer the questions themselves, with a minimum self-response rate of 80% for adults, and reproductive-age women were specifically required to answer relevant questions themselves. The follow-up compliance rate was required to be above 95%. if it fell short, a revisit to all surveyed households was conducted. Regarding questionnaire administration, there was a comprehensive promotion of electronic data collection methods, utilizing electronic tablet-based offline survey technology (Computer-Assisted Personal Interviewing System-CAPI) to replace traditional paper-based household surveys. The sampling method used in the Sixth National Health Service Survey was a multistage stratified cluster random sampling. In the first stage, counties (cities, districts) were sampled as the primary sampling units, with 10 socio-economic, cultural, educational, demographic, and health indicators related to health being selected through expert consultation and stepwise regression. The second stage involved sampling townships (streets), and the third stage involved sampling villages (neighborhood committees).

### The data

The cross-sectional data utilized in this study were extracted from the national household health service survey conducted by the CNHSS Survey. conducted in September 2018, were utilized. A total of 156 sample counties in urban areas were meticulously selected. This selection encompassed the choice of 5 townships (streets) from each sample county (urban area), 2 villages from each street, and 60 households from each village. Trained and qualified surveyors, utilizing the family health questionnaire form developed by the Chinese National Health Commission, conducted household visits to question the entire resident population within the sampled survey households. The content of the household health inquiry survey comprehensively covered demographic and socioeconomic characteristics of the survey respondents, aspects of health security, health service utilization, among other pertinent variables.

### Study population and measures

Aligning with the research objectives, the dataset was stratified according to the health insurance coverage status of respondents, following the definitions provided in a prior publication [25]. The cohort covered by UEBMI comprised 60,176 individuals,among whom 7581 possessed PHI, while 52,595 did not. The URBMI cohort consisted of 62,598 individuals, with 11,385 holding PHI and 51,213 lacking PHI.Furthermore, the NRCMS encompassed 125,421 individuals, including 14,502 with PHI and 110,919 without PHI.

Healthcare utilization metrics encompassed outpatient visits and inpatient admissions. Outpatient visits were defined as the proportion of household members seeking outpatient care at any facility within the 2 weeks preceding the survey. Inpatient admissions denoted the proportion of household members hospitalized in the year preceding the survey. Health care seeking behavior was evaluated through the distribution of outpatient visits and inpatient admissions, with health care providers categorized into clinics, outpatient departments, village clinics and township health centers in rural counties, as well as community health centers (stations) in urban cities, county hospitals, municipal hospitals, provincial and above hospitals, private hospitals, etc. Notably, clinics, outpatient departments, village clinics, and township health centers in rural counties, as well as community health centers (stations) in urban cities, were classified as primary care facilities.

### Theoretical model

The Andersen model is a mainstream framework in the healthcare sector for analyzing the factors influencing individual medical behavior and accessibility [26]. Over time, the model has undergone several revisions and adjustments, eventually stabilizing to include environment, population characteristics, health behaviorandoutcomes of care. According to the Andersen model, medical behavior is influenced by propensity characteristics, enabling resources, health service demands, the healthcare system, and outcomes of care [27–29]. This study adjusts the model based on data availability and research objectives, categorizing factors influencing the choice of healthcare institutions into propensity characteristics, enabling resources, and health service demands. Propensity characteristics include gender, age, marital status, and ethnicity. Enabling resources encompass health insurance type, urban or rural status, region, education level, employment status, average annual household income, and accessibility of healthcare services. Health service demands include whether an individual has a chronic condition and their perceived severity of illness. For each qualitative indicator, such as gender, marital status, education level, employment status, and chronic illness status, as well as for continuous indicators like age, the study categorizes and assigns dummy variables for analysis (Appendix 2).

### Statistical analysis

Outpatient visits and inpatient admissions represent dichotomous dependent variables with binary outcomes (yes or no). Binary logistic regression models were employed to investigate the impact of PHI, considering both its presence and absence, while controlling for other relevant variables. The binary logistic regression model is formulated as follows:

$$y_i\:=\:\:log\:it\;\left(p\right)\:=\:\delta_{\mathit0}\mathit\:\mathit+\mathit\:\delta_{\mathit1}\mathit\;X_i\:+\:\eta_{1i},\eta_{1i}\sim N\left(0,\:1\right)$$

where “ *y*_*i*_ ” is the probability of individual i seeking outpatient or inpatient care; *X*_*i*_ refers to explanatory variables, with or without PHI as the major independent variable in this study. *δ*_1_ is the parameter estimation of explanatory variables. *η*_1*i*_ are the residuals, *η*_1*i*_ ~ *N*(0, 1).

The dependent variables,pertaining to the health care provider, exhibited a categorical nature with more than two values. Health care providers were categorized into four distinct classes: 1 = primary care facilities, 2 = county hospitals, 3 = municipal hospitals, 4 = provincial and above hospitals. Multinomial logistic regression models (MNL) were employed to investigate the impact of PHI, considering both its presence and absence, while simultaneously controlling for other independent variables. The MNL models utilized in this study are delineated as follows:

$$\Pr \left(\textrm{y}=1\right)=\frac{e^{x\beta (1)}}{e^{x\beta (1)}+{e}^{x\beta (2)}+{e}^{x\beta (3)}+{e}^{x\beta (4)}}$$

$$\Pr \left(\textrm{y}=2\right)=\frac{e^{x\beta (2)}}{e^{x\beta (1)}+{e}^{x\beta (2)}+{e}^{x\beta (3)}+{e}^{x\beta (4)}}$$

$$\Pr \left(\textrm{y}=3\right)=\frac{e^{x\beta (3)}}{e^{x\beta (1)}+{e}^{x\beta (2)}+{e}^{x\beta (3)}+{e}^{x\beta (4)}}$$

$$\Pr \left(\textrm{y}=4\right)=\frac{e^{x\beta (4)}}{e^{x\beta (1)}+{e}^{x\beta (2)}+{e}^{x\beta (3)}+{e}^{x\beta (4)}}$$

The coefficients associated with the reference category are regarded as zero, and in this investigation, *β*_1_ = 0. Consequently, the aforementioned equations can be expressed as:

$$\Pr \left(\textrm{y}=1\right)=\frac{1}{1+{e}^{x\beta (2)}+{e}^{x\beta (3)}+{e}^{x\beta (4)}}$$

$$\Pr \left(\textrm{y}=2\right)=\frac{e^{x\beta (2)}}{1+{e}^{x\beta (2)}+{e}^{x\beta (3)}+{e}^{x\beta (4)}}$$

$$\Pr \left(\textrm{y}=3\right)=\frac{e^{x\beta (3)}}{1+{e}^{x\beta (2)}+{e}^{x\beta (3)}+{e}^{x\beta (4)}}$$

$$\Pr \left(\textrm{y}=4\right)=\frac{e^{x\beta (4)}}{1+{e}^{x\beta (2)}+{e}^{x\beta (3)}+{e}^{x\beta (4)}}$$

Concerning the reference category (*y* = 1), the relative probability of the second category (*y* = 2) is:

$$\frac{\Pr \left(y=2\right)}{\Pr \left(y=1\right)}={e}^{x\beta (2)}$$

This ratio is commonly denoted as the relative risk ratio (RRR), where X represents a vector of independent variables (*x*_1_, *x*_2_, *x*_3, ……_, *x*_*k*_), and *β*(2) is a vector comprising the calculated coefficients (*β*_1_(2), *β*_2_(2), ……, *β*_*k*_(2)). The RRR for one unit change of *Xi* is *e*^*βi*(2)^.

Within the present investigation, the RRR pertaining to the PHI variable elucidates the relative likelihood of respondents with PHI opting for a specific category of healthcare providers relative to the reference category. An RRR greater than 1 signifies an escalation in the probability, while an RRR less than 1 indicates a reduction in the probability. The statistical analyses were conducted using SAS software version 9.4. Significant values and 95% Confidence Intervals (CIs) for percentages were reported.

## Results

### Characteristics of study participants

In the UEBMI cohort, there exists a higher proportion of female individuals who have acquired, whereas in the URBMI and NRCMS cohorts, a greater prevalence of male individuals has purchased PHI. Regarding age distribution among PHI purchasers,the UEBMI group predominantly comprises young and middle-aged individuals, the URBMI group predominantly includes children and teenagers, and the NRCMS group exhibits a concentration of children, teenagers, and middle-aged individuals. The proportion of the elderly individuals is comparatively limited.Ethnically, across both PHI and non-PHI subgroups within the three basic medical insurance categories, the predominant demographic is Han Chinese (Table 1).

**Table 1:** Characteristics of the UEBMI, URBMI, NRCMS respondents with or without PHI

	UEBMI			URBMI			NRCMS		
	Total	With PHI	Without PHI	Total	With PHI	Without PHI	Total	With PHI	Without PHI
**Respondents numbers**	60,176	7581	52,595	62,598	11,385	51,213	125,421	14,502	110,919
**Gender**									
Male	51.4	46.1	52.2	47.3	50.3	52.6	49.3	52.3	46.9
Female	48.6	53.9	47.8	52.7	49.7	47.4	50.7	47.7	53.1
**Age**									
< 5	0.2	0.4	0.2	9.1	13.7	0.2	6	6.6	8.5
5–14	0.3	0.6	0.3	17.8	36	0.2	13.1	26.8	15.4
15–24	2.5	2.7	2.5	7.5	8	2.5	6.1	6.9	7.4
25–34	15.1	20	14.4	8.3	6.6	14.0	9.2	7.2	8.5
35–44	17.4	28	15.9	10.5	10.1	15.0	10.8	11.2	10.6
45–54	19.7	25.6	18.8	16.9	13.4	18.4	20.4	20.7	17.4
55–64	20.3	15.7	21.0	14.4	7.6	21.3	16.6	12.4	15.3
65+	24.6	7	27.1	15.5	4.6	28.5	17.8	8.1	16.9
**Folk**									
Han	95.5	95.8	95.5	89.5	90.3	95.4	86	93	89.4
Non-han	4.5	4.2	4.5	10.5	9.7	4.6	14	7	10.6
**Marital status (age 15+)**									
Not Married	7.1	7.6	7.0	13.3	18.3	7.0	9.2	11.4	12.6
Married	84.7	87.6	84.3	76.1	76.2	84.1	81.8	83.3	76.1
Widowed	5.7	2.1	6.2	8.1	3.7	6.5	7.6	4.1	8.7
Divorced	2.4	2.7	2.4	2.3	1.8	2.3	1.3	1.1	2.4
Other	0.2	0.1	0.2	0.2	0.1	0.2	0.2	0.1	0.2
**Education (age 15+)**									
None	2.0	0.4	2.2	11.2	5.7	2.4	18.2	11.9	11.9
Primary	9.3	3.8	10.1	25.5	16.8	10.5	33.2	25.5	26.6
Junior High	26.0	16.0	27.4	33.0	32.0	28.2	34.0	39.0	33.1
Senior High	19.0	18.1	19.1	16.1	23.0	19.2	9.2	15.3	15.2
Technical School	8.7	8.4	8.7	4.4	6.0	8.8	2.2	3.3	4.2
Junior college	18.0	26.0	16.8	5.9	10.0	16.2	2.2	3.5	5.4
College	15.1	24.3	13.8	3.3	5.8	13.1	0.8	1.2	3.0
Postgraduate	1.5	3.1	1.3	0.2	0.4	1.1	0.1	0.1	0.2
**Employment (age15+)**									
Employed	53.7	74.6	50.7	48.7	58.1	49.1	61.4	68.0	47.5
Retired	42.4	21.4	45.4	10.0	5.7	47.1	1.4	0.7	10.6
Student	0.2	0.2	0.2	7.1	13.4	0.2	3.8	8.3	6.3
Unemployed	3.7	3.8	3.7	34.2	22.8	3.7	33.6	23.0	35.7

Data are presented as percentage (%) unless otherwise stated

Within the demographic aged 15 and above, the predominant marital status among individuals covered by the three fundamental medical insurance schemes, both with and without PHI is married,with the unmarried demographic constituting the second largest group. In the URBMI group with PHI, a notably higher proportion is observed among the unmarried demographic. The educational distribution among individuals with PHI varies significantly across the three medical insurance schemes.Specifically, within the subsets of individuals with high school, technical school education or above, the percentages of those with PHI in the UEBMI, URBMI, and NRCMS groups were 79.4, 45.2, and 23.4%, respectively. Conversely, the educational distinctions among individuals without PHI in the UEBMI and URBMI groups were relatively less disparate, at 59.7 and 58.4%, respectively, while the proportion of individuals without PHI in the NRCMS group was lower, at 28%.

The predominant employment status across all six categories of medical insurance is characterized by active employment. Notably, the proportion of the unemployed demographic is relatively higher among individuals without PHI for the NRCMS at 32.1%. Conversely, among those with PHI for both NRCMS (21.5%) and the URBMI (19.7%), the unemployed population is comparatively lower. In contrast, the unemployed segments for the Urban Employee-Based Basic Medical Insurance (UEBMI), both with and without PHI, as well as those without PHI for participants across all schemes, exhibit lower proportions, ranging from 2.3% (Table 1).

### Healthcare utilization and provider choice

**Fig. 1:**
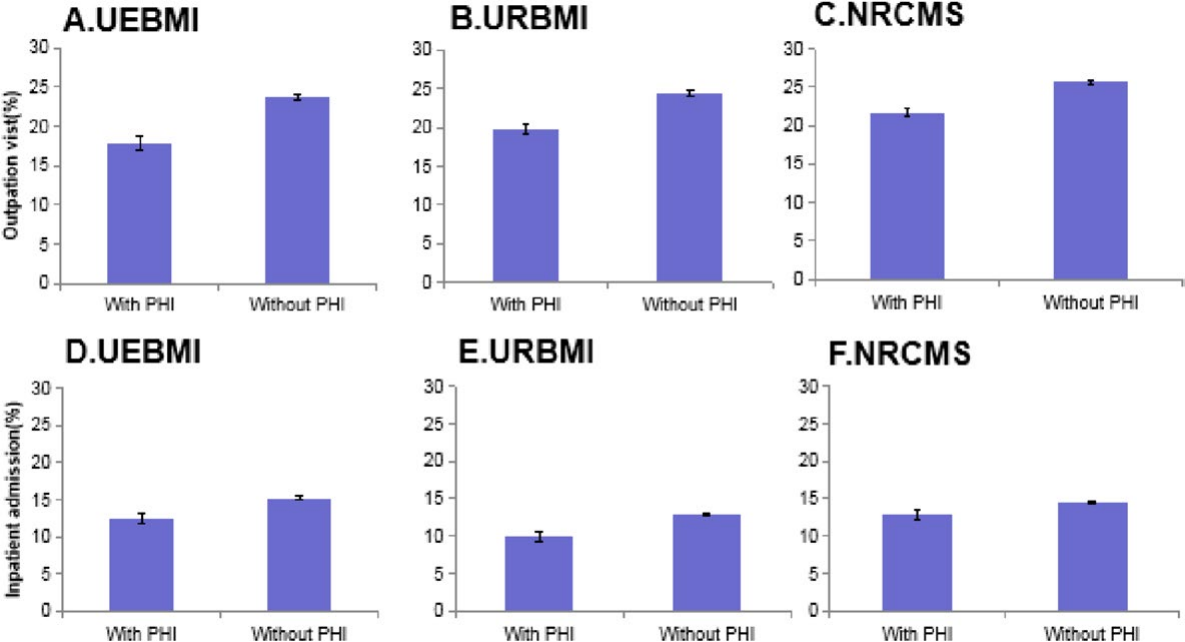
Healthcare utilization with 95%CI among respondents with and without PHI covered by UEBMI, URBMI, NRCMS. Notes: Panel **A**, **B**, and **C** shows outpatient visits of respondents with and without PHI covered by UEBMI, URBMI, and NRCMS, respectively. Panel **D**, **E**, and **F** shows inpatient admissions of respondents with and without PHI covered by UEBMI, URBMI, and NRCMS, respectively

Figure 1 illustrates the patterns of outpatient visits and inpatient admissions among respondents covered by UEBMI, URBMI, and NRCMS, differentiated by the presence or absence of PHI. In the case of UEBMI participants, a comparison between those with and without PHI reveals outpatient visit rates of 17.9 and 23.6%, and inpatient admission rates of 12.4 and 15.2%, respectively. Among URBMI participants, those with PHI exhibit outpatient visit rates of 19.8%, compared to 24.3% for those without PHI, with inpatient admission rates of 9.9 and 12.8%, respectively. Similarly, for NRCMS participants, those with PHI show outpatient visit rates of 21.7%, as opposed to 25.6% for those without PHI, accompanied by inpatient admission rates of 12.9 and 14.5%, respectively. Notably, both outpatient visits and inpatient admissions are observed to be lower among individuals with PHI in the context of these three basic medical insurance schemes, in comparison to those without PHI.

**Fig. 2:**
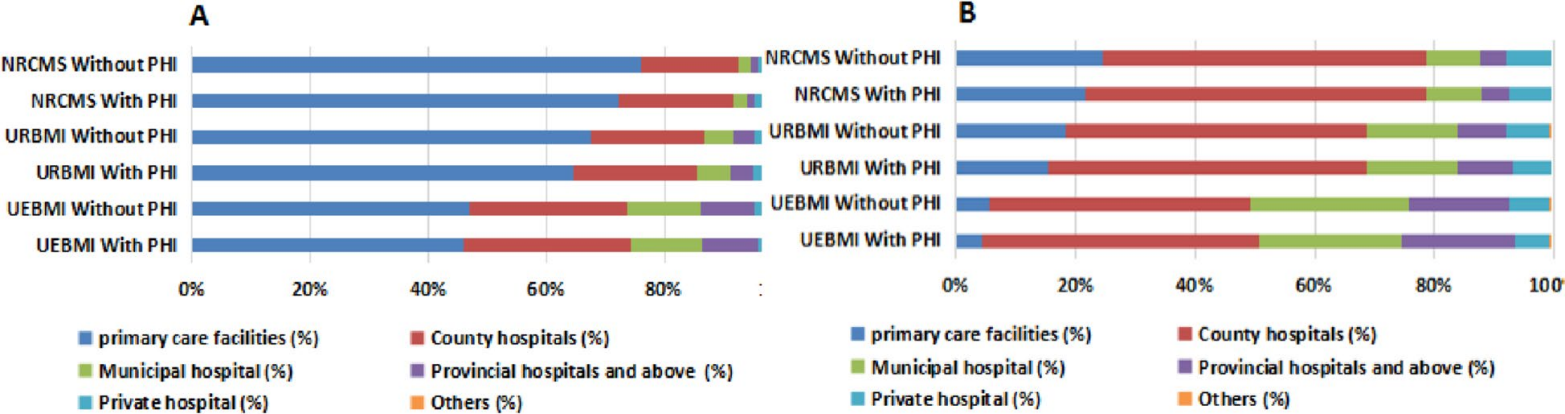
Distribution of outpatient visits (**A**) and inpatient admissions (**B**) by healthcare provider choice. Notes: Primary care facilities contain clinics, outpatient departments, village clinics and township health centers in rural counties, and community health centers (stations) in urban cities

Figure 2 presents the distributions of outpatient visits and inpatient admissions based on healthcare provider choices among respondents covered by UEBMI, URBMI, and NRCMS stratified by the presence or absence of PHI. A higher proportion of individuals covered by UEBMI with PHI opt for primary care facilities as their initial healthcare visit, contrasting with those covered by UEBMI without PHI (45.9% vs. 42.9%). In contrast, both groups with PHI under URBMI (64.6%) and NRCMS (72.2%) exhibit lower proportions of individuals selecting primary care facilities for their first visit in comparison to their counterparts without PHI under URBMI (67.4%) and NRCMS (76.0%). Regarding inpatient admissions, the proportions of individuals choosing primary care facilities are lower across all groups with PHI covered by UEBMI (4.5%), URBMI (15.4%), and NRCMS (21.6%) compared to their respective groups without PHI covered by UEBMI (5.5%), URBMI (18.2%), and NRCMS (24.5%).

### Impact of PHI on healthcare utilization

Table 2 delineates the influence of supplementary PHI on healthcare utilization patterns among respondents encompassed by UEBMI, URBMI, and NRCMS. Controlling for the variables related to propensity characteristics, enabling resources and health service needs, the probability of outpatient visits and inpatient admissions were higher among UEBMI participants with PHI in comparison to their counterparts without PHI (*p* < 0.05). Conversely, for URBMI participants, The probability of outpatient visits and inpatient admissions were higher among those with PHI,although the differences did not attain statistical significance. In the case of NRCMS participants, the probability of outpatient visits and inpatient admissions was lower for individuals with PHI compared to those without PHI.However, the probability of inpatient admissions was higher among those with PHI, and these discrepancies reached statistical significance (*p* < 0.05).

**Table 2:** Impact of PHI on healthcare utilization among respondents covered by UEBMI, URBMI, and NRCMS

	Outpatient visit			Inpatient admission		
	ß	P	OR	ß	P	OR
UEBMI						
with PHI	0.0952	0.0153	1.100(1.018,1.188)	0.139	0.001	1.149(1.058,1.249)
URBMI						
with PHI	0.0095	0.8239	1.010(0.928,1.098)	0.0797	0.0955	1.083(0.986,1.189)
NRCMS						
with PHI	−0.1019	0.001	0.903(0.850,0.960)	0.206	< 0.0001	1.229(1.152,1.311)

*OR* odds ratio. In outpatient visit models, variables controlled include gender, age, marital status, folk, urban/rural residence, region, education, employment, household annual income per capita groups, with catastrophic health insurance or not, with chronic diseases or not, distance to the nearest healthcare provider, and self-feeled severity of the illness. In inpatient admission models, variables controlled include gender, age, marital status, folk, urban/rural residence, region, education, employment, household annual income per capita groups, with catastrophic health insurance or not, with chronic diseases or not, and distance to the nearest healthcare provider

### Impact of PHI on healthcare provider choice

Table 3 presents the influence of PHI on the selection of healthcare providers among respondents encompassed by UEBMI, URBMI, and NRCMS. Following adjustments for variables associated with propensity characteristics, enabling resources, and health service needs, NRCMS participants with PHI exhibited a heightened likelihood of opting for a higher level hospital in comparison to those without PHI. The relative probability of choosing county hospitals and provincial and above hospitals was notably 17% higher (Relative Risk, RR = 1.17, 95% CI, 1.03–1.34) and 27% higher (RR = 1.27, 95% CI, 1.26–1.28), respectively, for individuals with PHI than for those without medical insurance, and these differences reached statistical significance. Similarly, UEBMI and URBMI participants with PHI displayed a greater tendency than their counterparts without PHI to select higher-ranked hospitals for consultations. However, none of these distinctions achieved statistical significance.

**Table 3:** Impact of PHI on choice of healthcare provider among respondents covered by UEBMI, URBMI, and NRCMS

	County hospitals			Municipal hospitals			Provincial and above hospitals		
	ß	P	RRR	ß	P	RRR	ß	P	RRR
A. Outpatient visit									
UEBMI									
with PHI	0.1734	0.0504	1.19(1,1.415)	0.0545	0.6603	1.06(0.828,1.347)	0.2012	0.1447	1.22(0.933,1.602)
URBMI									
with PHI	0.011	0.9044	1.01(0.845,1.209)	−0.1944	0.283	0.82(0.577,1.174)	0.0326	0.8785	1.03(0.68,1.57)
NRCMS									
with PHI	0.1581	0.0179	1.17(1.028,1.335)	0.0052	0.1779	1.01(0.998,1.013)	0.2400	< 0.0001	1.27(1.26,1.283)
B. Inpatient admission									
UEBMI									
with PHI	0.0077	0.9658	1.01(0.71,1.43)	−0.1074	0.5748	0.9(0.617,1.307)	0.1335	0.4985	1.14(0.776,1.682)
URBMI									
with PHI	0.1626	0.1619	1.18(0.937,1.478)	−0.2672	0.139	0.77(0.537,1.091)	0.0613	0.7708	1.06(0.704,1.606)
NRCMS									
with PHI	0.1073	0.1211	1.11(0.972,1.275)	−0.0351	0.7551	0.97(0.774,1.204)	0.0932	0.5245	1.1(0.824,1.463)

*RRR* relative risk ratio. Variables controlled include gender, age, marital status, folk, urban/rural residence, region, education, employment, household annual income per capita, with catastrophic health insurance or not, with chronic diseases or not, and distance to the nearest healthcare provider

After accounting for variables related to propensity characteristics, enabling resources, and health service demands, individuals with PHI within the three distinct basic health insurance cohorts demonstrated a heightened likelihood of hospitalization in county hospitals and provincial and above hospitals as opposed to primary care facilities. Meanwhile, the probability of hospitalization in municipal hospitals decreased, although none of these observed distinctions reached statistical significance (*p* > 0.05).

## Discussion

This investigation, utilizing binomial logistic regression and multinomial logistic regression models on 2018 CNHSS data, sheds light on the influence of PHI on the outpatient and inpatient service utilization, as well as health care provider choice of individuals covered by basic medical insurance schemes.

Binomial logistic regression findings indicate that UEBMI participants with PHI exhibit elevated probabilities of outpatient visits and inpatient admissions compared to those without PHI. This suggests that the presence of dual health insurance, to a certain extent, promotes the utilization of health care services, increases the probability of residents seeking medical treatment, and promotes residents to seek medical treatment without delay. These results align with previous studies by international scholars David Cantarero-Prieto, Sara Moreira, et al. [11–14] and chinese scholars such as Chai Huamin [15] and Yang Liu [30]. However, despite the observed significance, the correlation appears relatively weak. This weak association may be attributed to the lower expenditure payouts by PHI companies, leading residents with lower incomes and high out-of-pocket expenses to opt against seeking medical treatment. The limited participation of PHI in China’s health insurance system is evident in the low proportion of PHI claims in the country’s social health expenditure and total health costs. In 2018, PHI premium claims expenditure accounted for 6.8% of social health expenditure and 3.0% of total health costs [31], indicating its constrained role. Data from the China Insurance Yearbook and the China Health Statistics Yearbook reveals that the proportion of PHI claims expenditure in total health costs fluctuated between 1.9 and 3.6% from 2015 to 2019, remaining below 4.0%. Presently, PHI in China primarily relies on individual insurance, imposing a substantial financial burden. Consequently, differentiated PHI policies or interventions for these different groups should be made, such as the design of different PHI products and adjustments in insurance reimbursement rates to improve healthcare accessibility and quality. However, these results are inconsistent with those of Bo Hai [17] and Chen Hua [2]. The reason may be related to the smaller sample size of individuals with commercial insurance in the CHARLS data they used, which could affect estimated effects.

Additionally, the findings indicate that among urban residents enrolled in social health insurance, those with PHI have higher probabilities of outpatient visits and hospital admissions compared to those without commercial insurance, although the differences are not statistically significant. This phenomenon may be related to characteristics of the population and features of the insurance plans. The higher likelihood of outpatient visits and hospital admissions among the insured group with PHI coverage could be due to PHI offering more medical choices and higher medical compensation, thus increasing the likelihood of seeking healthcare service. To further promote the rational use of healthcare service and improve the level of financial risk protection, the government should take measures such as encouraging the innovation and development of PHI products, strengthening the level of primary healthcare services, and improving the accessibility and quality of healthcare services, to ensure that more people can access to effective health care with reasonable quality and financial risk protection.

Furthermore, the findings reveal a decreased probability of outpatient visits and an increased likelihood of inpatient admissions among NRCMS participants with PHI compared to those without PHI. This implies that the acquisition of PHI by NRCMS patients positively stimulates the demand for hospitalization, augmenting the health demand for patients who refrain from hospitalization. This observation aligns with Yang Liu’s research, which underscores that the burden reduction effect of NRCMS primarily manifests in outpatient medical services, while PHI predominantly influences inpatient medical care [30]. Similarly, Luo Jingxian [32] notes that, given the low reimbursement ratio of hospitalization expenses under NRCMS, PHI exerts a more pronounced negative impact on hospitalization costs, leading residents to exhibit a relatively higher propensity to choose hospitalization for their illnesses. This inclination can be attributed to the prevalent types of PHI available in the market, which largely concentrate on hospitalization insurance formats, such as fixed benefit insurance for major diseases, compensatory insurance for hospitalization medical expenses, and hospitalization allowances [33]. For rural patients predominantly covered by NRCMS, engaging in PHI can mitigate the share of out-of-pocket inpatient expenses, concurrently enhancing the accessibility of medical and health services for NRCMS patients. Consequently, the outcomes of this study offer valuable insights for decision-makers in addressing health needs. Policy makers should encourage commercial insurers to develop more PHI products targeting outpatient care through taxation and other policies to provide supplemental financial risk protect for outpatient care needs.

The outcomes of the multinomial logistic regression analysis reveal that the presence of PHI significantly increases the likelihood of NRCMS participants opting for county hospitals and provincial and above hospitals. This enhancement contributes, to a certain extent, to improved access to high-quality healthcare for the PHI insured, thereby mitigating the disparities in healthcare utilization between urban and rural areas. This finding aligns with the research conducted by international scholars Rodríguez M,Jones A. M,et al. [18–21] and chinese scholars Luo Jingxian [32]. However, for UEBMI and URBMI participants with PHI, the results indicate a higher inclination toward selecting higher-level hospitals compared to those without PHI, although this difference lacks statistical significance.

Such phenomena may be related not only to population characteristics and insurance plan features but also to the quality of healthcare institutions and the inability of medical insurance companies to comprehensively cover medical institutions for treatment. For example, reimbursement requirements in PHI contracts often impose certain limitations on healthcare institutions, while healthcare provided by community health service centers, township health centers, and similar institutions often do not meet the reimbursement criteria. This can result in medical insurance companies failing to comprehensively cover medical institution visits. In light of these observations, On one hand, it is recommended to augment the value proposition of PHI to enhance the patient consultation experience and boost satisfaction. This may involve expanding patients’ rights to select medical institutions in pursuit of higher-quality medical services and improved comfort, aligning with models observed in countries like Australia and the United Kingdom. In Australia, individuals can voluntarily engage in PHI, facilitating expedited access to medical services when needed, and concurrently allowing the freedom to choose healthcare providers for enhanced medical services [34]. On the other hand, it is suggested to improve the contracts and service networks of PHI. Enhancing the provisions of PHI contracts can ensure coverage for primary healthcare institutions, enabling policyholders to enjoy reimbursement benefits when seeking healthcare. Establishing a sound medical service network ensures healthcare coverage in both urban and rural areas, including primary healthcare institutions, to reduce residents’ reliance on tertiary hospitals and improve the coverage and accessibility of medical services [35]. However, the findings of this study are inconsistent with those of Buchmueller, Schokkaert, et al. [22–24]. The reason for this discrepancy may be the differences in healthcare systems and insurance frameworks across different regions or countries, which could also play a significant role.

Additionally, the study concludes that, in comparison to UEBMI and URBMI, the synergy between NRCMS and PHI is more harmonious, with government policies more supportive of this combination. This assertion corresponds with findings in existing literature, such as those by Chai Huamin [15] and Luo Jingxian [32], both empirically demonstrating a more pronounced impact of PHI on NRCMS patients, signifying a substantially higher policy effect of PHI in rural areas compared to urban areas.

This study is subject to several limitations that warrant acknowledgment. Firstly, the reliance on self-reported information in the CNHSS data, gathered through household surveys, introduces an inherent susceptibility to reporting bias or recall bias. While rigorous quality control measures were implemented throughout the survey stages, the potential for biases cannot be entirely eliminated. Furthermore, despite the national representativeness of the CNHSS survey sample, the lack of available data on the characteristics of UEBMI, URBMI, and NRCMS populations in current public datasets precludes definitive confirmation that the survey respondents accurately represent their corresponding target populations. Secondly, the adoption of a cross-sectional research design poses challenges in addressing endogenous issues related to private health insurance. This design limitation constrains the ability to establish causal relationships between PHI variables and healthcare utilization, as well as provider choice variables. The study recognizes the presence of variations in health status, service accessibility, and utilization preferences among diverse insurance-covered populations, with the potential for unaccounted or inadequately measured confounding factors. Despite efforts to control for confounding effects through multivariables modeling analysis, the comprehensive capture of all potential confounding elements remains challenging.Thirdly, this investigation primarily adopts a demand-side perspective to assess the impact of PHI on healthcare utilization and medical institution choice. However, it overlooks the influence of PHI on the supply-side behavior of medical service providers. In reality, the introduction of PHI may induce changes in the treatment behavior of medical service suppliers, potentially exerting a more substantial impact on the utilization and provision of medical services than that observed on the demand side.

## Conclusion

The findings of this investigation reveal nuanced variations in the impact of supplementary PHI on health care utilization and seeking behavior across distinct populations covered by diverse social health insurance schemes. UEBMI participants exhibit heightened utilization of outpatient and inpatient services associated with PHI. In the case of NRCMS participants, PHI is correlated with a preference for higher-level hospital outpatient visits and increased utilization of inpatient care. Conversely, URBMI participants do not manifest any significant effects on health care utilization and choice. To advance the development of PHI and enhance the multi-tiered social security system, it is imperative to implement diverse measures. Recommendations for the government include the exploration of policy instruments such as tax incentives, premium rebates, and surcharges tailored to PHI participants within various social health insurance frameworks. Additionally, insurance companies are advised to intensify efforts in product innovation and differentiated rate structures. These initiatives can potentially broaden the reach of PHI, augmenting medical service accessibility, and substantially fortifying the overall welfare protection afforded to individuals.

### Supplementary Information

**Supplementary Material 1.**

## Data Availability

No datasets were generated or analysed during the current study.

## Abbreviations

PHI: private health insurance
SHI: social health insurance
CNHSS: China National Health Services Survey
UEBMI: urban employee-based basic medical insurance
URBMI: urban resident-based basic medical insurance
NRCMS: new rural cooperative medical scheme
MNL: multinomial logistic regression

## References

[1] The Ministry of Health (2012). China health statistical Yearbook-2012.

[2] Chen H, Yang X (2022). Research on adverse selection and moral Hazard of rural residents participating in commercial medical insurance. Rural Econ..

[3] Jung HW, Kwon YD, Noh JW. How public and private health insurance coverage mitigates catastrophic health expenditures in Republic of Korea. BMC Health Serv Res. 2022;22(1):1042. 10.1186/s12913-022-08405-4. 10.1186/s12913-022-08405-4 PMC9377807 35971176

[4] Sekhri N, Savedoff W (2005). Private health insurance: implications for developing countries. Bull World Health Organ..

[5] State Administration of Financial Supervision.Table of Original Insurance Premium Income by Region of the Country. 2008,2019. http://www.cbirc.gov.cn/cn/view/pages/tongjishuju/tongjishuju.html. Accessed 16 Jul 2023.

[6] Center for Health Statistics and Information NHC (2021). An analysis report of National Health Service Survey in China, 2018.

[7] Yu mingren,Chenyu,Xujuan.Current status of the development of universal health insurance at the provincial level in China. Chinese health Resources,2022,25(02):217–222+229.

[8] Kiil A, Arendt JN (2017). The effect of complementary private health insurance on the use of health care services. Int J Health Econ Manag..

[9] Jowett M, Deolalikar A, Martinsson P (2004). Health insurance and treatment seeking behaviour: evidence from a low-income country. Health Econ..

[10] Choi Y, Kim JH, Yoo KB (2015). The effect of cost-sharing in private health insurance on the utilization of health care services between private insurance purchasers and non-purchasers: a study of the Korean health panel survey (2008-2012). BMC Health Serv Res..

[11] Cantarero-Prieto D, Pascual-Sáez M, Gonzalez-Prieto N (2017). Effect of having private health insurance on the use of health care services: the case of Spain. BMC Health Serv Res..

[12] Moreira S, Barros PP. Double health insurance coverage and health care utilization: evidence from quantile regression. Health Econ. 2010;19(9):1075–92. 10.1002/hec.1641. 10.1002/hec.1641 20730998

[13] Barros PP, Machado MP, Sanz-de-Galdeano A (2008). Moral hazard and the demand for health services: a matching estimator approach. J Health Econ..

[14] Franc C, Perronnin M, Pierre A (2016). Supplemental health insurance and healthcare consumption-a dynamic approach to moral Hazard. Health Econ..

[15] Chai H (2013). An empirical analysis of urban and rural residents' demand for medical services and medical insurance in China. World Econ Pap..

[16] Fu C. The Effect of Commercial Medical Insurance under Conditions of Social Basic Medical Insurance-from the Perspective of Medical Expenditure. Anhui University of Finance and Economics, 2022. https://link.cnki.net/doi/10.26916/d.cnki.gahcc.2022.000185. 10.26916/d.cnki.gahcc.2022.000185. [in Chinese].

[17] Bo H, Zhang YA (2015). Study on the Adverse Selection of Supplemental Commercial medical Insurance-Evidence Based on CHARLS Data. Insurance Stud..

[18] Rodríguez M, Stoyanova A (2004). The effect of private insurance access on the choice of GP/specialist and public/private provider in Spain. Health Econ..

[19] Jones AM, Koolman X, Doorslaerb EV (2006). The impact of supplementary private health insurance on the use of specialists in European countries. Annales d'économie et de Statist..

[20] González-Álvarez L, Clavero A (2009). Inequalities in health care utilization in Spain due to double insurance coverage. An Oaxaca-ransom decomposition. Soc Sci Med..

[21] Vera‐Hernández ÁM. Duplicate coverage and demand for health care. The case of Catalonia. Health economics, 1999;8(7):579-598. 10.1002/(sici)1099-1050(199911)8:7<579::aid-hec478>3.0.co;2-p 10544325

[22] Buchmueller TC, Couffinhal A, Grignon M (2004). Access to physician services: does supplemental insurance matter? Evidence from France. Health Econ..

[23] Schokkaert E, Ourti TV, Graeve DD (2010). Supplemental health insurance and equality of access in Belgium. Health Econ..

[24] Jonneke Bolhaar, Maarten Lindeboom, Bas van der Klaauw,. A dynamic analysis of the demand for health insurance and health care. European Economic Review, 2012;56:669–690. 10.1016/j.euroecorev.2012.03.002.

[25] Yan X, Liu Y, Rao K (2022). Trends in disparities in healthcare utilisation between and within health insurances in China between 2008 and 2018: a repeated cross-sectional study. Int J Equity Health..

[26] Li Y, Lu S (2017). Theoretical construction and analysis path evolution of the Andersen model. Chinese health. Serv Manag..

[27] Andersen MR (2008). National health surveys and the behavioral model of health services use. Med Care..

[28] Kehrer B, Andersen R, Glaser WA (1972). A behavioral model of families' use of health services. J Hum Resour..

[29] Lemming MR, Calsyn RJ (2004). Utility of the behavioral model in predicting service utilization by individuals suffering from severe mental illness and homelessness. Community Ment Health J..

[30] Yang L. The effect of commercial medical insurance under conditions of new rural cooperative Medical System.Yunnan University, 2016. https://kns.cnki.net/kcms2/article/abstract?v=YoFA4grnCX5xhYGFt3m7DNPS2xPDOau9JylB51P6MEGEf6SEEdCjUh3Zj7_9v_Ww7ZGyXz1PzZtrA7MV9LRRg5bWv2GQJPxTNjtFAFd7kgF28K3BABv2dLprRAa76TiLZVTWkg5iY0hxL3954bB4sQ==&. [in Chinese].

[31] State Administration of Financial Supervision. Table of Original Insurance Premium Income by Region of the Country. 2019. http://www.cbirc.gov.cn/cn/view/pages/ItemDetail.html?docId=371349&itemId=954&generaltype=0. Accessed 16 Jul 2023.

[32] Luo J (2022). The impact of commercial medical insurance on urban and rural medical service utilization differences.Southwest.

[33] Odeyemi I, Nixon J. Assessing equity in health care through the national health insurance schemes of Nigeria and Ghana: a review-based comparative analysis. Int J Equity Health. 2013;12:1-18. 10.1186/1475-9276-12-9. 10.1186/1475-9276-12-9 PMC3626627 23339606

[34] Australian Government Deparment of Health and Aged Care. About private health insurance. 2021. https://www.health.gov.au/health-topics/privatehealthinsurance/aboutprivatehealthinsurance. Accessed 15 Jul 2023.

[35] Van Hees SGM, O’Fallon T, Hofker M, et al. Leaving no one behind? Social inclusion of health insurance in low-and middle-income countries: a systematic review. Int J Equity Health. 2019;18:1-19. 10.1186/s12939-019-1040-0. 10.1186/s12939-019-1040-0 PMC6714392 31462303

