# Supplementary material for: Does supplemental private health insurance impact health care utilization and seeking behavior of residents covered by social health insurance? Evidence from China National Health Services Survey
Source: Int J Equity Health. 2024 May 31;23:113. doi: 10.1186/s12939-024-02158-8 (PMC11143651; doi:10.1186/s12939-024-02158-8)
Supplement: Supplementary file 1 — Supplementary Material 1. [file 12939_2024_2158_MOESM1_ESM.pdf]

Appendix 1.Development history of private health insurance in China

With the deepening of China's health care reform policy, the PHI policy has also developed and innovated, going through the initial stage, professional exploration stage and innovative development stage. In 1982, the People's Insurance Company of China started the "Shanghai Cooperative Employees' Medical Insurance" on a pilot basis. 1995, after the introduction of the Insurance Law of the People's Republic of China, Chinese PHI companies were established one after another, and individual additional term critical illness insurance was first introduced. In 1998, the Decision of the State Council on the Establishment of the UEBMI proposed that "medical expenses exceeding the maximum payment limit can be settled through PHI" and "the establishment of supplementary medical insurance for enterprises is allowed". Since 2000, developed countries have gradually introduced supplementary medical insurance, that is private medical insurance, and combined public medical insurance with private insurance in the medical system. At the same time, China's PHI as supplementary medical insurance has shown a rapid development trend, with the premium income of health insurance and its share in personal insurance increasing year by year, with premiums increasing nearly 11 times and the share increasing by 17.4 percentage points from 2011 to 2020. Several Chinese policies also provide a good institutional environment for the development of PHI, positioning it as a supplement to basic health insurance.

With the approval of the China Insurance Regulatory Commission(CIRC) in 2004 to establish professional PHI companies such as health insurance, the state of

PHI being dependent on life insurance and property and casualty insurance was changed, and the era of professional exploration began. In 2006, the CIRC promulgated the "Measures for the Administration of Health Insurance", the first regulation on the specialization of health insurance in China, which unified the regulatory rules for business entities and clarified the regulatory requirements for the operation of PHI. In 2009, the "Opinions of the Central Committee of the Communist Party of China and the State Council on Deepening the Reform of the Medical and Health System" explicitly proposed to accelerate the establishment and perfection of a system that focuses on basic medical insurance, other forms of supplementary medical insurance and PHI. On August 10, 2014, the State Council issued the Opinions on Accelerating the Development of Modern Insurance Service Industry, proposing to "establish PHI as an important pillar of the social security system" and to "give full play to the importance of PHI". In October 2016, the Central Committee of the Communist Party of China and the State Council issued the "Health China 2030" planning outline, pointing out that by 2030 the modern PHI service industry will be further developed, and the proportion of PHI payouts to total health costs will be significantly increased. In December of the same year, the State Council issued the "Thirteenth Five-Year Plan" to deepen the reform of the medical and health system, which proposed to "promote the development of PHI" as one of the five major initiatives to establish an efficient operation.

The establishment of China's National Health Insurance Administration in 2018 marked the further deepening of payer reform, giving the health insurance

administration more responsibilities and putting more demands on health insurance management. In January 2020, the China Banking and Insurance Regulatory Commission(CBIRC) and 13 other departments jointly issued the "Opinions on Promoting the Development of Private Health Insurance in Social Services", which proposed that "health insurance products and services will be improved ", "accelerate the approval and filing of exclusive life insurance products for the elderly aged 60 and above", "encourage insurance institutions to provide comprehensive protection services for medical treatment, illness, care and maternity", and "To support PHI institutions to participate in medical insurance services and medical insurance cost control, and cooperate with medical insurance institutions.". In February 2020, the Central Committee of the Communist Party of China and the State Council issued the "Opinions on Deepening the Reform of the Medical Security System" also clearly proposed to promote the development of a multi-level medical security system and accelerate the development of PHI. On March 5, 2020 ,The State Council issued the Opinions on Deepening the Reform of the Medical Security System, requiring efforts to address the unbalanced and inadequate development of medical security, enrich the supply of PHI products, improve the level of protection for serious diseases and diversified medical needs, and explore the mechanism to protect the use of drugs for rare diseases, establishing a direction for universal supplementary medical insurance from the top-level design. The Notice on Regulating the Business of Urban Customized Private Health Insurance of Insurance Companies issued by the General Office of the CBIRC on June 8, 2021 defines the term of "inclusive health insurance"

as "urban customized private health insurance". As of October 15, 2021, 19 of China's 31 provincial-level administrative regions have launched a total of 26 provincial-level universal health insurance policies, with coverage focused on inpatient expenses and special drug expenses within the medical insurance catalog. Beijing Universal Health Insurance, for example, is a standardized universal health insurance policy jointly directed by the Beijing Municipal Bureau of Medical Insurance and the Beijing Municipal Bureau of Local Financial Supervision, and supervised by the Beijing Supervision Bureau of the China Banking and Insurance Regulatory Commission, and is offered by five insurance companies: PICC, State Life Insurance, Taikang Pension, China Pacific Life, and Ping An Insurance. It has the following features: the coverage is broad. It can pay for medical expenses within the medical insurance catalog, medical expenses outside the medical insurance catalog, and medical expenses incurred for the purchase of high cost special drugs; the requirements for participation are low. There are no specific requirements for age, occupation, gender, etc., and coverage is available for people with specific preexisting conditions. Until December 29, 2022, 3 million people have enrolled Beijing Universal Health Insurance. In addition to the "Beijing Universal Health Insurance", the more popular universal insurance for residents include the Million Dollar Medical Insurance and the City Customized Private Health Insurance. This shows that the acceptance of PHI by the population is gradually increasing. The history of the Chinese supplemental PHI is as follows.

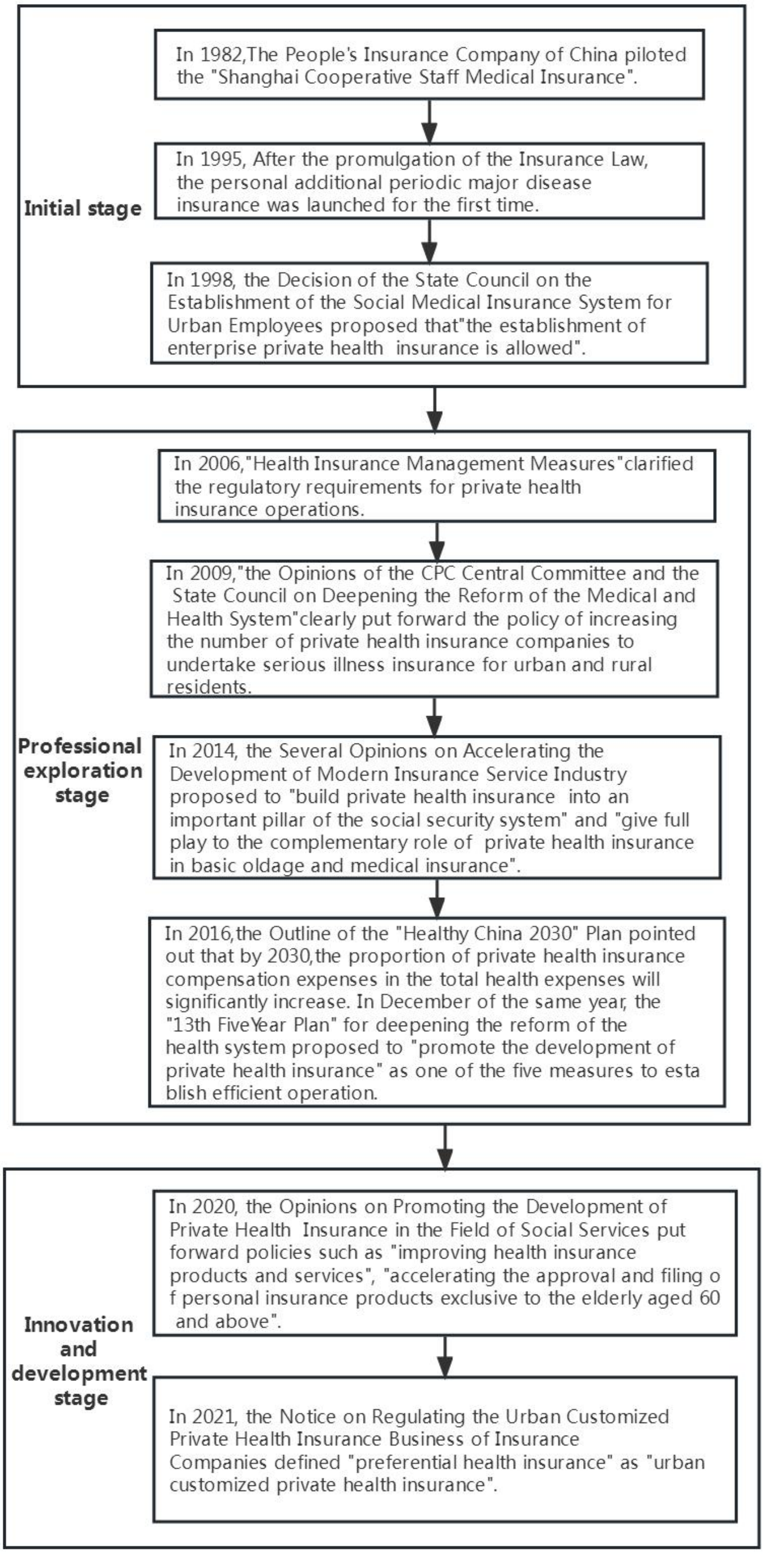

Figure A: Development history of private health insurance in China

Appendix 2.assignment of explanatory variables in the multi-factor regression model

Table 1 assignment of explanatory variables in the multi-factor regression model

Dimension	Variable Name	Assignment	
Propensity characteristics	Gender	1 Male	0 Female
	Age group (control = 15-)		
	25-	1 Yes	0 No
	35-	1 Yes	0 No
	45-	1 Yes	0 No
	55-	1 Yes	0 No
	65 years old and above	1 Yes	0 No
	Marital status (control = unmarried)		
	Married	1 Yes	0 No
	Divorce	1 Yes	0 No
	Bereaved spouse	1 Yes	0 No
	Folk	1 Han Chinese	0 Non-Han Chinese
Enabling resources	Region (control = east)		
	Middle	1 Yes	0 No
	West	1 Yes	0 No
	Northeast	1 Yes	0 No
	Education level (control = no schooling)		
	Primary School	1 Yes	0 No
	Junior High School	1 Yes	0 No
	High School	1 Yes	0 No
	Mechanic/Secondary	1 Yes	0 No
	College and above	1 Yes	0 No
	Employment status (control = active)		
	Retirement	1 Yes	0 No
	Current Students	1 Yes	0 No
	Unemployed or jobless	1 Yes	0 No
	Type of medical insurance (control =new rural cooperative medical scheme)		
	Urban resident-based basic medical insurance	1 Yes	0 No
	Urban resident-based basic medical insurance	1 Yes	0 No
	Pure PHI	1 Yes	0 No
	Without medical insurance	1 Yes	0 No
	PHI	1 Yes	0 No
	Major medical insurance	1 Yes	0 No
	Per capita income (control = lowest income group)		
	Lower income group	1 Yes	0 No
	Middle Income Group	1 Yes	0 No

Dimension	Variable Name	Assignment	
Health service demands	Higher income group	1 Yes	0 No
	Top Income Group	1 Yes	0 No
	Presence of chronic diseases (control = none)	1 Yes	0 No
	The severity of one's own feeling of illness and injury (control = not serious)		
	General	1 Yes	0 No
	Serious	1 Yes	0 No
